# Death due to delirium: a case of a self-cut hemodialysis dialysis catheter - a case report

**DOI:** 10.1186/s12882-019-1571-z

**Published:** 2019-10-28

**Authors:** Pierre Antoine Brown, Peter O. Magner, Swapnil Hiremath, Edward G. Clark

**Affiliations:** 1 Department of Medicine, University of Ottawa, Ottawa, Ontario Canada; 2 Division of Nephrology, Department of Medicine, University of Ottawa, 1967 Riverside Dr, Ottawa, Ontario K1H 7W9 Canada; 3 Kidney Research Centre, Ottawa Hospital Research Institute, Ottawa, Ontario Canada

**Keywords:** Delirium, End stage kidney disease (ESKD), Peritoneal Dialysis, Peritonitis, Self-harm

## Abstract

**Background:**

Neuropsychiatric conditions such as depression, delirium and cognitive impairment are common in patients with end-stage kidney disease (ESKD) and individuals suffering from ESKD are more likely to commit suicide than members of the general population. Self-harm gestures are not infrequent for ESKD patients suffering from depression, but not well described in other conditions.

**Case presentation:**

We present a case of self-harm in a patient with ESKD suffering from acute delirium. A man in his mid-seventies was admitted with fungal peritoneal dialysis (PD) associated peritonitis. On the first day post operatively, he was found with absent vital signs due to exsanguination from newly inserted central catheter which he which had self-severed. He died a few days later as a result of the self-harm gesture.

**Conclusion:**

This case highlights that delirium may lead to self-harm events in ESKD and identifies a few strategies to help reduce the risk of self-harm events.

## Background

Neuropsychiatric conditions such as depression, delirium and cognitive impairment are common in patients with end-stage kidney disease (ESKD) [1, 2]. Individuals suffering from ESKD are more likely to commit suicide than members of the general population [3, 4] and self-harm gestures are relatively common in depressed patients receiving dialysis [3, 5]. Less is known about the consequences of delirium in the ESKD population [4]. Here, we report the case of a hospitalized patient with ESKD who died as result of delirium. His death occurred after a self-inflicted gesture: he cut his own hemodialysis central venous catheter (CVC) with a pair of nail scissors.

## Case presentation

We present the case of a male patient, in his mid-70s, with ESKD secondary to diabetic nephropathy on peritoneal dialysis (PD) for 18 months prior to being admitted to hospital. His past medical history included type-2 diabetes, emphysema, bronchiectasis and a remote ischemic stroke that left him with minor motor deficits. He relied on support from his spouse to set up his PD but was reported to have been enjoying a good quality of life prior to his admission.

Six months prior to his admission, he had developed PD-associated peritonitis due to coagulase negative staphylococcus. He completed a course of intraperitoneal (IP) vancomycin with complete resolution of symptoms. Three weeks prior to his admission, he again developed abdominal pain and a cloudy dialysis effluent and was diagnosed with recurrent peritonitis secondary to coagulase negative staphylococcus. Vancomycin therapy lead to a good initial clinical response, but a few weeks later he presented with a relapse of his symptoms which led to his admission. *Candida albicans* was cultured from the PD effluent. Within 12 h of his admission, he underwent an uncomplicated surgical removal of his peritoneal dialysis catheter and insertion of a tunneled central venous catheter (CVC) for transition to hemodialysis. Post-operatively, he was noted by the nurses to be in stable condition and mentating normally. His first hemodialysis treatment in hospital was uneventful. The following night, his nurses documented mild confusion which was not commented upon at the change of shift the following morning (post-admission day (PAD) 3). At that time, the patient requested his toiletry bag (which he had brought from home), which was provided to him. The contents of his bag were not examined by the caring team, as this was not part of the standard admission checklist to a medical ward.

Less than an hour later, the patient was found collapsed in his hospital room washroom. He was naked and surrounded by a large pool of blood. Immediate help was requested and vital signs were not detected, ACLS procedures were initiated. During CPR, it was noted that the patient was bleeding profusely in the area of his chest. Under a dressing, he had a cleanly severed CVC with approximately 5 cm of catheter still protruding from the chest wall. This was immediately clamped. With ACLS interventions, he regained a pulse and a blood pressure after ~ 15 min. He was transferred to the intensive care unit (ICU).

**Fig. 1:**
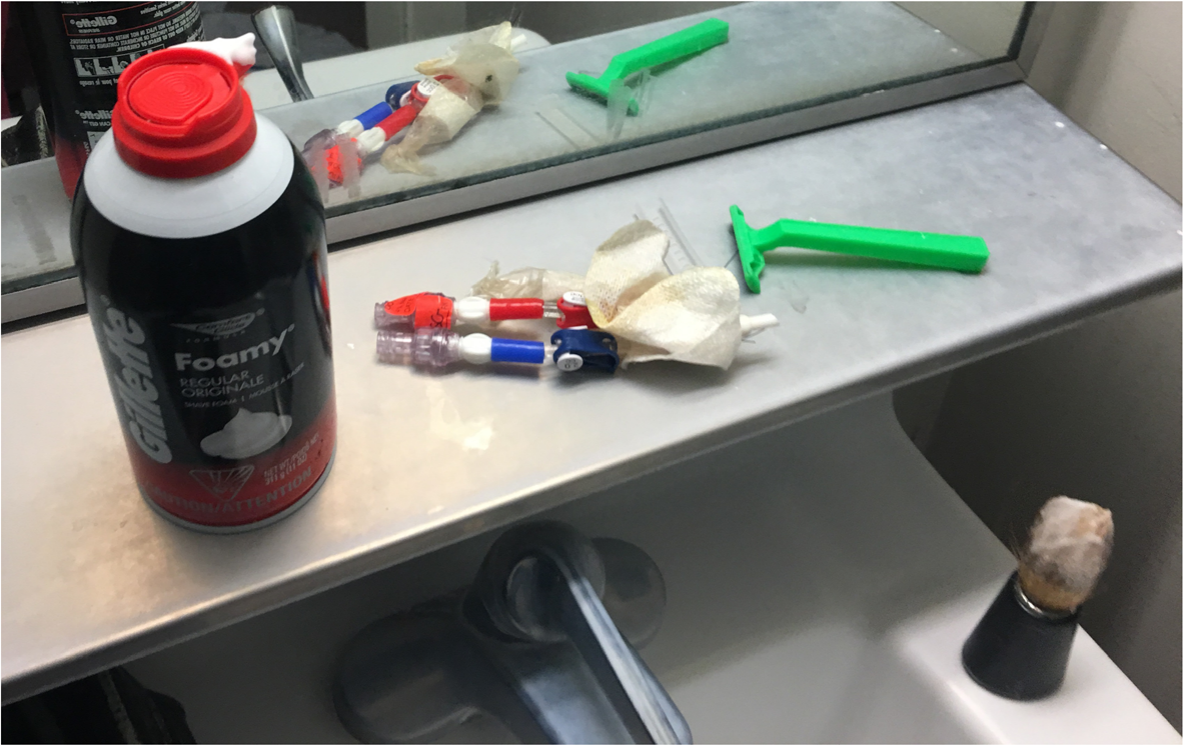
Severed remaining portion of patient’s CVC

**Fig. 2:**
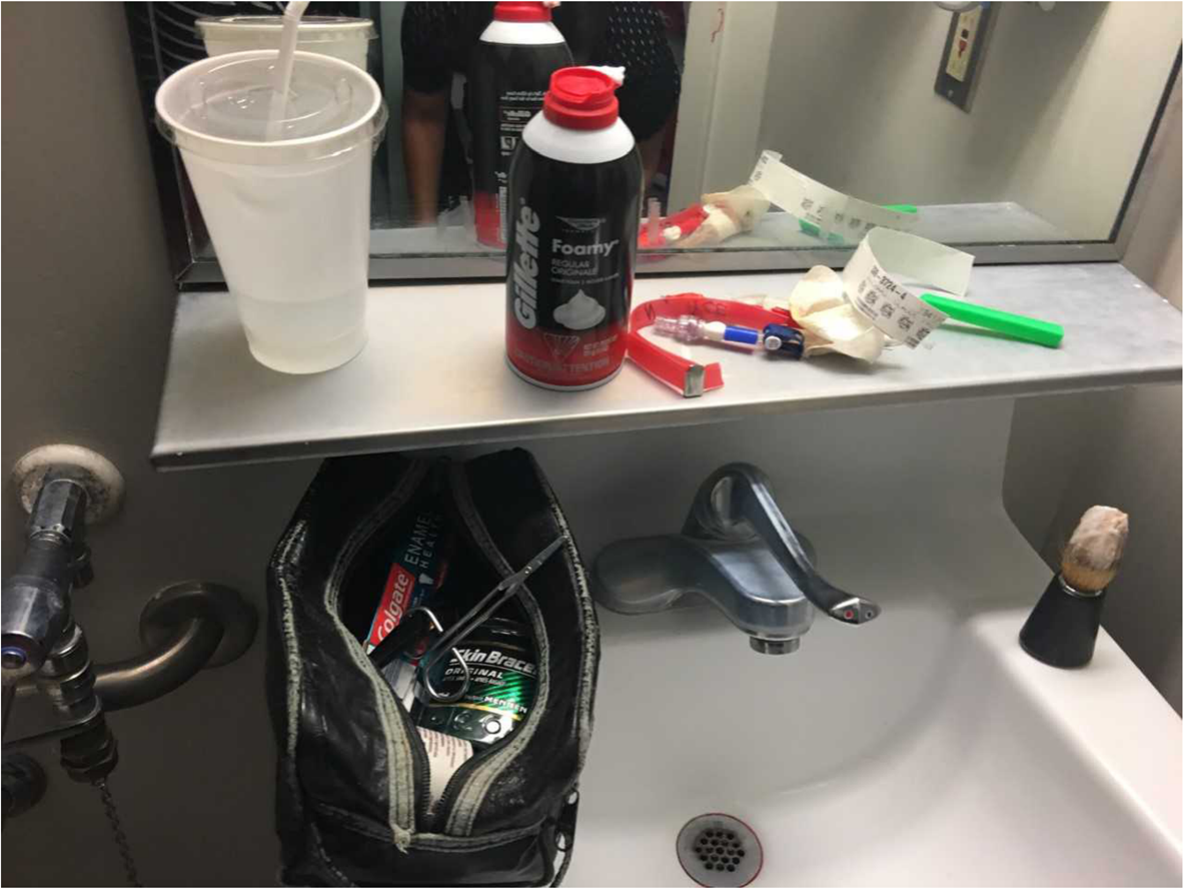
Severed patient’s bracelet and toiletry bag containing scissors

In his washroom, a bloody gown, the remaining portion of his CVC (Fig. 1), as well as his severed patient identification bracelet was found. His toiletry bag included small, sharp scissors (Fig. 2).

In the context of his underlying medical issues, his condition worsened in the ICU over the following 3 days. After a family meeting to review the patient’s goals of care on PAD 6, his goals of care were changed to focus on comfort, he passed away soon after.

## Discussion and conclusions

This patient’s untimely demise was precipitated by catastrophic exsanguination due to what could be perceived as a self-harm gesture. Death by exsanguination as a result of a self-harm gesture to a hemodialysis access has been previously been reported in the context of depression but not, to our knowledge, delirium. Edirisinghe and Busutil (2006) reported that a 79 year-old man with depression died after severing his subclavian catheter [6]. Mizukami et al. (2010) reported the case of a 48 year-old women who exsanguinated after intentionally opening the lumen of her CVC [7]. Charlot and deRoux (2009) reported on a chronic hemodialysis patient with a history of depression and suicidal ideation who bit into his forearm arteriovenous graft and died from exsanguination [8]. Finally, Marc et al. (2000) reported a suicide by way of a self-inflicted stab wound of the arteriovenous fistula for hemodialysis access in an elderly woman [9]. Nonetheless, suicide by this manner is appears to be uncommon. Gill et al. (2012) reported a case series of 100 deaths due to hemorrhage from hemodialysis arteriovenous fistulas and grafts (not CVCs) in which only 2 cases were certified as suicides [10].

Unique aspects of the case that we report are that our patient did not have any history of depression. However, no routine screening for depression was in place in our program at the time of the events. While a detailed post-mortem interview with his spouse did not suggest any signs or symptoms of a major depressive episode or cognitive impairment prior to his admission, it is impossible to definitely exclude depression in his case as no formal screening was ever conducted. He did, however, develop mild confusion after undergoing surgery and CVC insertion which likely represented delirium. Thus, this report highlights delirium as a likely cause of potentially preventable self-induced injuries by way of damage to the hemodialysis vascular access.

While delirium is common in the ESKD population [1] and more likely to occur in elderly patients hospitalized with an acute medical illness [11], it is often under recognized and difficult to diagnose and treat [12]. Irrespective, some preventative measures might have helped minimize the risk of self-harm in this case. First, routine screening for sub-clinical depression in ESRD patients could have potentially led to the diagnosis and treatment of depression if it was present in this case. In the US, the Centers for Medicare & Medicaid Services (CMS) has made routine depression screening & treatment a standard quality measure for all dialysis facilities [13]. In Ontario, routine screening for depression is now achieved via the comprehensive Edmonton Symptom Assessment System Revised: renal, which is now done routinely in all dialysis units. Second, institutions can develop policies and procedures to minimize the risk of self-harm in all patients at high risk of delirium. This can include checklists that mandate verification of personal items sharp objects (such as scissors, razors and nail clippers) that could be used for self-harm and implementation of standardized delirium assessment tools in patients at high risk [14]. Other strategies could include more frequent visual assessments of patients at risk or suspected of having delirium. Third, awareness to the increased risk of self-harm in patients with depression and delirium through continuous education might lead to better vigilance by front line personnel such as nurses and health care aids. At our institution’s nephrology ward, new policies were developed. All sharp items (e.g. suture scissors, scalpel blades, large bore needles) are now kept under supervised access only. As our patient appears to have used scissors found in his own toiletry bag to have severed his CVC, a policy that allows screening of a patient’s belongings is being examined.

In conclusion, hemodialysis accesses can present a deadly opportunity for self-harm. While events of this nature are rare, they may be underreported due to the stigma that might be associated with them. While such events have previously been reported in patients with documented depression, there is significant overlap of depression, delirium and impaired cognition in the CKD population [1]. Our case highlights that, presumably unintentional, self-harm can occur in the context of delirium alone. As such, clinicians and institutions should have a low threshold to take measures that will reduce the risk of either intentional or inadvertent harm to a patient’s own dialysis access.

## Data Availability

Not applicable.

## Abbreviations

ACLS: Advanced cardiac life support
CPR: Cardiopulmonary resuscitation
CVC: Central venous catheter
ESKD: End-stage kidney disease
ICU: Intensive care unit
IP: Intraperitoneal
PAD: Post admission day
PD: Peritoneal dialysis
